# Early Fractional Amplitude of Low Frequency Fluctuation Can Predict the Efficacy of Transcutaneous Auricular Vagus Nerve Stimulation Treatment for Migraine Without Aura

**DOI:** 10.3389/fnmol.2022.778139

**Published:** 2022-02-24

**Authors:** Menghan Feng, Yue Zhang, Zeying Wen, Xiaoyan Hou, Yongsong Ye, Chengwei Fu, Wenting Luo, Bo Liu

**Affiliations:** ^1^Department of Radiology, The Second Affiliated Hospital of Guangzhou University of Chinese Medicine, Guangzhou, China; ^2^The Second Clinical College, Guangzhou University of Chinese Medicine, Guangzhou, China; ^3^Department of Radiology, The First Affiliated Hospital of Henan University of Chinese Medicine, Zhengzhou, China

**Keywords:** migraine without aura (MWoA), transcutaneous auricular vagus nerve stimulation (taVNS), fractional amplitude of low frequency fluctuation (fALFF), functional magnetic resonance imaging (fMRI), support vector regression (SVR)

## Abstract

Migraine is a common primary headache disorder. Transcutaneous auricular vagus nerve stimulation (taVNS) has been verified to be effective in patients with migraine without aura (MWoA). However, there are large interindividual differences in patients’ responses to taVNS. This study aimed to explore whether pretreatment fractional amplitude of low frequency fluctuation (fALFF) features could predict clinical outcomes in MWoA patients after 4-week taVNS. Sixty MWoA patients and sixty well-matched healthy controls (HCs) were recruited, and migraineurs received 4-week taVNS treatment. Resting-state functional magnetic resonance imaging (rs-fMRI) data were collected, and the significant differences of fALFF were detected between MWoA patients and HCs using two-sample *t*-test. A mask of these significant regions was generated and used for subsequent analysis. The abnormal fALFF in the mask was used to predict taVNS efficacy for MWoA using a support vector regression (SVR) model combining with feature select of weight based on the LIBSVM toolbox. We found that (1) compared with HCs, MWoA patients exhibited increased fALFF in the left thalamus, left inferior parietal gyrus (IPG), bilateral precentral gyrus (PreCG), right postcentral gyrus (PoCG), and bilateral supplementary motor areas (SMAs), but decreased in the bilateral precuneus and left superior frontal gyrus (SFG)/medial prefrontal cortex (mPFC); (2) after 4-week taVNS treatment, the fALFF values significantly decreased in these brain regions based on the pretreatment comparison. Importantly, the decreased fALFF in the bilateral precuneus was positively associated with the reduction in the attack times (*r* = 0.357, *p* = 0.005, Bonferroni correction, 0.05/5), whereas the reduced fALFF in the right PoCG was negatively associated with reduced visual analog scale (VAS) scores (*r* = −0.267, *p* = 0.039, uncorrected); (3) the SVR model exhibited a good performance for prediction (*r* = 0.411, *p* < 0.001),which suggests that these extracted fALFF features could be used as reliable biomarkers to predict the treatment response of taVNS for MWoA patients. This study demonstrated that the baseline fALFF features have good potential for predicting individualized treatment response of taVNS in MWoA patients, and those weight brain areas are mainly involved in the thalamocortical (TC) circuits, default mode network (DMN), and descending pain modulation system (DPMS). This will contribute to well understanding the mechanism of taVNS in treating MWoA patients and may help to screen ideal patients who respond well to taVNS treatment.

## Introduction

Migraine, a common chronic neurological disorder, is characterized by recurrent headache and typically accompanied by nausea, photophobia, and sensitivities to light–sound–smell (Steiner et al., 2013). Migraine without aura (MWoA) subtype is the most prevalent type, which accounts for nearly 70% of the total (Rasmussen and Olesen, 1992). Currently, medication therapy for MWoA could provide pain control at 45 min to 48 h and easily lead to addiction and other adverse effects (Diener et al., 2012; Lanteri-Minet, 2014; Westergaard et al., 2014; Abu Bakar et al., 2016; Buse et al., 2016). Transcutaneous auricular vagus nerve stimulation (taVNS), one kind of non-invasive neuromodulation technique, has been verified to relieve headache intensity and reduce the frequency of migraine attacks for MWoA patients in several clinical trials (Luo W. et al., 2020; Zhang et al., 2021). Notably, despite the effectiveness of taVNS for MWoA, the efficacy varies considerably across different subjects. Therefore, identifying a valid and objective biomarker for treatment response will be of great importance as it could help screen ideal migraineurs to improve the clinical efficacy and avoid the waste of medical resources.

Resting-state functional magnetic resonance imaging (rs-fMRI) is an emerging non-invasive imaging technique, which could be used to identify brain areas of the aberrant functional activities through measuring the spontaneous brain activity by low-frequency fluctuations in blood oxygen level-dependent (BOLD) signals (Biswal et al., 1995; Guo et al., 2011; Liu et al., 2012). Currently, the common analysis methods of fMRI data include the fractional amplitude of low-frequency fluctuation (fALFF), regional homogeneity (ReHo), and functional connectivity (FC). However, ReHo is easily affected by some parameters such as the magnitude of spatial smoothing (Zang et al., 2004) and is insensitive to shape differences (Cole et al., 2010). As for FC, it focuses on the whole functional activity of the brain but is dependent on the user-defined region of interests (ROIs) based on the prior knowledge (Cole et al., 2010; Smitha et al., 2017). As known, fALFF is an index reflecting the intensity of spontaneous neuronal activity in local brain regions, and more neuroimaging studies applied fALFF to explore the underlying pathophysiology mechanism of different diseases, including in MWoA (Xue et al., 2013; Wang et al., 2016; Li et al., 2017; Hu et al., 2019). Researchers have demonstrated that taVNS could regulate the disrupted brain functional activities in MWoA patients (Zhang et al., 2019a; Luo W. et al., 2020), which provides a new perspective to reveal the neural mechanism of treatment. In addition, fALFF has been proved to be more objective and sensitive in prediction studies (Sui et al., 2018; Li et al., 2020). To sum up, we used the fALFF to perform the analysis of brain function in this study. However, currently, conventional fMRI studies were mainly based on univariate and group-level statistical methods, few is known about whether the altered fALFF could be used in the prediction of an individual patient with MWoA.

Under the limited translational applicability of standard mass-univariate analytical methods that are typically used in neuroimaging, a great hope is given to a data-driven multivariate machine learning technique—multivariate pattern analysis (MVPA), which is sensitive to the fine-grained spatial discriminative patterns and exploration of inherent multivariate nature from high-dimensional neuroimaging data. Previous studies have widely applied MVPA in the classification or prediction of individual treatment response (Redlich et al., 2016; Cash et al., 2019; Tu et al., 2019; Messina and Filippi, 2020; Yin et al., 2020; Yu et al., 2020). For example, one recent study applied MVPA to identify the useful biomarkers of the FC between the medial prefrontal cortex (mPFC) and specific subcortical regions, which could significantly predict the changes in symptoms in patients with chronic low back pain receiving 4-week acupuncture treatment (Tu et al., 2019). Similarly, Hou et al. (2016) employed multivariate analysis to construct a model for the prediction of Parkinson’s disease (PD) severity ratings from the baseline individual fMRI data. In addition, several researchers selected meaningful categorical features between migraine patients and healthy controls (HCs) as a region of interest (ROI) to predict the efficacy of acupuncture using a support vector regression (SVR) model (Tu et al., 2020).

Even though MVPA has been proved to be a promising approach in the application of predicting neurological disorders, to date, no literature has been published on the individual prediction of taVNS treatment for MWoA. In this study, we will explore the differences in resting-state brain activities between MWoA patients and well-matched HCs and further test the predictive ability of those baseline fALFFs as the biomarkers for the clinical outcomes of taVNS treatment in MWoA patients using SVR. Therefore, we proposed three hypotheses: (1) MWoA patients would be associated with altered activities in specific brain regions compared with HCs. We compared the fALFF differences between MWoA patients and HCs, and a mask of these significant abnormal regions was generated and used for the subsequent analysis. (2) We hypothesized that taVNS could treat the MWoA through modulating the abnormal fALFF of these regions. Then, we explored how the taVNS could modulate those abnormal fALFFs in patients with MWoA, through comparing the fALFF differences between pre- and posttreatment in the mask. (3)We further supposed that the abnormal fALFF of these regions in the mask at baseline could serve as a reliable biomarker to predict the taVNS treatment outcomes for MWoA patients using a SVR model combined with feature select of weight based on the LIBSVM toolbox. We hope that our study could provide a quantitative benchmark for selecting suitable MWoA patients for taVNS treatment.

## Materials and Methods

### Participants

In our previous published article (Zhang et al., 2021), the taVNS has been confirmed as an effective treatment for MWoA relieving acute pain [especially in reducing visual analog scale (VAS) scores] in migraine patients, and This study was an advanced exploration to predict the efficacy of taVNS based on the SVR algorithm. Sixty right-handed MWoA patients who received 4-week taVNS treatment and sixty age-, gender-, and education-level-matched healthy controls were recruited. The Research Ethics Committee of the Second Affiliated Hospital of Guangzhou University of Chinese Medicine approved the study. This study protocol was registered on the Chinese Clinical Trial Registry (ChiCTR-INR-17010559). Written informed consent was obtained from all participants.

Episodic migraineurs without aura were diagnosed by licensed neurologists according to the 2nd Edition International Classification of Headache Disorders for Migraine Without Aura (Headache Classification Subcommittee of the International Headache Society, 2004). The detailed inclusion criteria for the MWoA patients are as follows: (1) aged 18–45 years old, (2) right-handed, (3) have at least 6 months of migraine duration, (4) have at least two headache attacks per month, (5) have not taken any prophylactic headache medications during the past 1 month, and (6) have not taken any psychoactive or vasoactive drugs during the past 3 months. Excluded criteria include the following: (1) headache induced by other diseases, (2) headache attack within 48 h prior to the experiment or during the experiment, (3) pregnancy or lactation, (4) any other chronic pain conditions, (5) severe head deformity or intracranial lesions, (6) score on the Self-Rating Anxiety Scale (SAS) or the Self-Rating Depression Scale (SDS) > 50.

### Interventions

**FIGURE 1:**
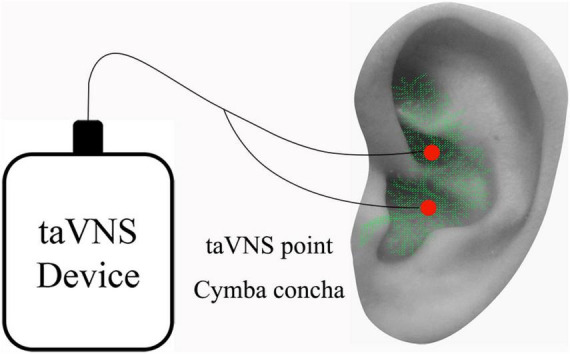
taVNS stimulation site on the left ear. Red dots indicate regions of taVNS (ABVN, green color). *ABVN*, auricular branch of the vagus nerve; *taVNS*, transcutaneous auricular vagus nerve stimulation.

For the patients with MWoA, we applied auricular vagus nerve electrical stimulation at the left cymba concha (Figure 1). The stimulation was applied with the MRI compatible electronic acupuncture treatment instrument (SDZII, Huatuo, Suzhou, China) by trained physicians. Similar to a previous taVNS study on migraine (Straube et al., 2015), we have chosen the frequency of 1 Hz with the duration of 0.2 ms. The stimulation was continuously applied for 30 min during each treatment session. Stimulation intensity was adjusted to approximately 1.5–5 mA, the strongest sensation that patients could tolerate without pain. All MWoA patients who were included in the final analysis completed a total of 12 treatment sessions during the 4-week treatment period.

### Clinical Outcome Measures

The treatment effect of tVNS for migraines has been confirmed in many literatures (Silberstein et al., 2016; Tassorelli et al., 2018; Diener et al., 2019). In our previous published article (Zhang et al., 2021), the taVNS also has been confirmed as an effective treatment for MWoA, and this study was an advanced exploration to predict the efficacy of taVNS based on the SVR algorithm. According to the results of Kinfe Thomas M study (Kinfe et al., 2015) and our previous study (Zhang et al., 2021), which suggest that tVNS can relieve acute pain (especially in reducing VAS scores) in migraine patients. So, we selected the improvement in VAS scores (post–pretreatment) as the primary outcome and the secondary outcome included attack times, total duration, Migraine-Specific Quality-of-Life Questionnaire (MSQ), Zung SDS, and Zung SAS in this study. In addition, according to the guidelines (Zheng, 2002), we take 25% reducing VAS scores as the judging criterion of the taVNS effective treatment.

The study lasted for 8 weeks: 4 weeks before the treatment (the baseline) and 4 weeks during the treatment. Patients were instructed to complete headache diary records after enrollment until this study finished. The diaries, MSQ, Zung SDS, and Zung SAS were collected at week four and week eight. The headache diary documented the onset time, duration, pain intensity (measured by VAS score), accompanying symptoms, and rescue medication use (ibuprofen suspension). To avoid prophylactic medication, we just selected occasional migraine patients, who tended to have a relatively low migraine attack frequency. Before the trial, ibuprofen suspension (H19991011, Shanghai Johnson and Johnson Pharmaceuticals, Ltd., 10 ml each time) was uniformly administered to MWoA patients according to the guidelines for diagnosis and treatment of migraine (Li et al., 2011). Patients were instructed not to use the medicine unless it is necessary. If the patient took the medication, the time, frequency, and dosage of each medication use would be recorded in the headache diary. In addition, patients were advised to forbid barbiturates, opiates, and prophylactic medication.

### Magnetic Resonance Imaging Data Acquisition

All rs-fMRI scanning was conducted on a 3.0T Siemens MRI scanner (Siemens MAGNETOM Verio 3.0T, Erlangen, Germany) with a 24-channel phased-array head coil. Subjects were told to stay awake, remain motionless, and keep their eyes closed during the scan. Tight, but comfortable, foam padding was used to minimize head motion, and earplugs were used to reduce scanner noise.

All patients participated in identical fMRI scanning sessions before and after 4 weeks of treatment. The scanning sessions include the 8-min resting-state fMRI scan and T1-weighted high-resolution structural images. rs-fMRI encompassing the whole brain was acquired in 8 min with a gradient-recalled echo-planar imaging pulse sequence and imaging parameters were as follows: repetition time (TR) = 2,000 ms, echo time (TE) = 30 ms, field of view (FOV) = 224 mm × 224 mm, matrix = 64 × 64, flip angle = 90°, slice thickness = 3.5 mm, interslice gap = 0.7 mm, 31 axial slices paralleled, and 240 time points. T1-weighted high-resolution structural images were applied with the following parameters: TR = 1,900 ms, TE = 2.27 ms, flip angle = 9°, FOV = 256 mm × 256 mm, matrix = 256 × 256, and slice thickness = 1.0 mm.

### Resting-State Functional Magnetic Resonance Imaging Data Processing

rs-fMRI data were preprocessed and analyzed using the SPM12^1^ and DPABI 3.0. The main steps included the following: (1) discarding the first 10 time points; (2) slice-timing correction, realignment, and discarding subjects with a mean framewise displacement value exceeding 0.5 mm or a maximum displacement greater than one voxel size (Power et al., 2012; Luo N. et al., 2020); (3) reorienting functional and T1 images with six rigid-body parameters; (4) coregistering T1 images to functional space, segmentation, and normalizing the functional images to Montreal Neurological Institute (MNI) space; (5) correcting head motion with Friston 24-parameter model (Friston et al., 1996; Yan et al., 2013), removing linear trend, and regressing out the white matter and cerebrospinal fluid signals; (6) resampling the functional images to 3 mm × 3 mm × 3 mm cubic voxels and smoothing functional images with a 6-mm Gaussian kernel of full width at half maximum; (7) temporally filtering (0.01–0.08 Hz) (Sun et al., 2021) to generate the ALFF value, and then, the fALFF map was obtained by dividing the total ALFF values from 0.01 to 0.025 Hz; and (8) transforming the fALFF map to the *z*-fALFF map with normal *z* transformation.

### Statistical Analysis

#### Statistical Analysis of Clinical Outcomes

Statistical analyses were performed with SPSS v20.0, and the significance threshold was set to *p* < 0.05 (two-tailed). Baseline demographic and clinical data were compared between MWoA patients and HCs using the chi-square test for categorical variables and the Student’s *t*-test for continuous ones (if normally distributed), and Mann–Whitney test (if not normally distributed). In addition, paired *t*-tests were employed to determine whether the alterations in clinical outcomes were significant after taVNS treatment for patients with MWoA, if characteristics were normally distributed and Wilcoxon signed rank test if not normally distributed.

#### Statistical Analysis of Functional Magnetic Resonance Imaging Data

The intergroup analysis (MWoA patients vs. HCs) of fALFF at baseline was applied using a two-sample *t*-test, with the age, sex, education level, and mean FD as covariates. A threshold of voxel-wise *p* < 0.005 uncorrected and a cluster-level *p* < 0.05 corrected by false discovery rate (FDR) were used for multiple comparison corrections between MWoA patients and HCs. The intragroup comparison of fALFF (pretreatment vs. posttreatment migraineurs) was performed using a paired *t*-test with the voxel-wise *p* < 0.001 uncorrected and cluster-level *p* < 0.05 family-wise error (FWE) corrected, and the mean FD and medication use dosage (ibuprofen suspension) were used as covariates. Moreover, to assess the association of neuroimaging findings with clinical outcomes, we performed a partial correlation analysis between pre- and posttreatment fALFF alterations and corresponding changed clinical variables (VAS score and attack times) after taVNS treatment for patients with MWoA, and Bonferroni correction was used for multiple comparisons.

#### Multivariate Pattern Analysis

This study applied MVPA method to explore whether the fALFF indicator was able to predict the treatment response of taVNS for patients with MWoA. We selected the abnormal regions, which indicate significant changes between migraine patients and HCs as our mask and proposed that these fALFF values at baseline would be used for predicting the taVNS treatment efficacy.

Here, MVPA based on linear SVR (implemented by LIBSVM^2^) was employed to verify our hypothesis. We set the changes in pain severity (VAS change) as the dependent variable and abnormal fALFF in the mask as independent variables (predictors) in all MWoA patients. A feature selection based on weight was used to reduce the data dimensions. The fixed 10-fold crossvalidation (CV) method was the compromising choice for bias and training sample size to avoid the risk of overfitting, with training sample and testing sample 9 and 1 part, respectively. We calculated the correlation coefficient of prediction-outcome correlation (r), which was defined as the correlation between the actual and predicted value, to evaluate the predictive ability of SVR model. To further assess the performance of the model and evaluate the significance of r, we ran permutation testing. In each test, we randomly permuted the labels of the data prior to training. Ten-fold CV was then performed on the permuted datasets, and the procedure was repeated 1,000 times to determine whether the performance occurred by chance, and *p* < 0.001 was considered to be statistically significant.

**FIGURE 2:**
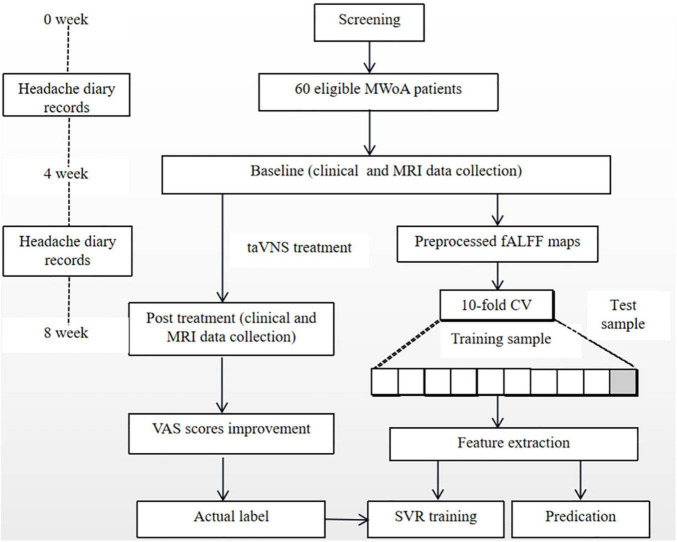
Flowchart of prediction analysis. *MWoA*, migraine without aura; *fALFF*, fractional amplitude of low-frequency fluctuation; *VAS*, visual analog scale; *CV*, crossvalidation; *SVR*, support vector regression; *taVNS*, transcutaneous auricular vagus nerve stimulation.

The flowchart of the prediction analyses is shown in Figure 2.

## Results

### Demographic Characteristics and Clinical Outcomes

In this study, a total of 60 right-handed MWoA patients (13 men and 47 women, age of 31.72 ± 6.65 years, 54 higher education and 6 lower than higher education) and also 60 gender-, age-, and education-level-matched HCs (15 men and 45 women, age of 29.25 ± 7.26 years, 53 higher education and 7 lower than higher education) finished this study.

Baseline demographic characteristics between MWoA patients and HCs were compared as follows. The age, as a continuous variable, was tested to be normally distributed. Additionally, the two-sample *t*-test result showed that compared with HCs, there was no significant difference (*p* > 0.05) in age in MWoA patients. We divided the education level into two subgroups, higher education and lower than higher education similar to Jasilionis’s study (Jasilionis and Shkolnikov, 2016). The chi-square test was used to compare the gender and education-level difference, and results indicated that the gender and education level of MWoA patients were not significantly different (*p* > 0.05), compared with HCs. The details are presented in Table 1.

**TABLE 1:** Demographic characteristics and clinical outcomes of MWoA and HCs.

	Item	HCs (*n* = 60)	MWoA (*n* = 60)			*P*
			Pretreatment	Posttreatment	Post–pre[95% CI]	
Gender	Male	15	13	13		0.666
	Female	45	47	47		
Education level	Lower than higher education	7	6	6		0.769
	Higher education	53	54	54		
Age (Years)		29.25 ± 7.26	31.72 ± 6.65	31.72 ± 6.65		0.060
VAS scores			49.1 ± 16.6	32.1 ± 20.5	17.0 [11.40; 22.50]	<0.001
Attack times			3.4 ± 2.2	2.5 ± 1.9	0.90 [0.30; 1.50]	0.005
Total duration			61.5 ± 69.2	29.1 ± 39.4	32.4 [14.50; 50.30]	0.001
MSQ			58.6 ± 10.6	71.6 ± 9.3	−13.0 [−16.10; −9.97]	<0.001
SDS			46.6 ± 8.6	43.3 ± 8.8	3.30 [1.60; 5.10]	<0.001
SAS			45.3 ± 9.56	41.6 ± 8.03	3.66 [1.69; 5.64]	<0.001

*VAS, visual analog scale**;** MSQ, Migraine-Specific Quality-of-Life Questionnaire; SAS, Self-Rating Anxiety Scale; SDS, Self-Rating Anxiety Scale; CI (confidence interval. The p-values were obtained by paired or two-sample t-test or chi-square test.*

Clinical outcomes between pre- and posttreatment MWoA patients were compared as follows. The continuous variables (VAS scores, attack times, total duration, MSQ, SDS, and SAS) were tested to be normally distributed, and the paired *t*-test results showed significant improvement in clinical outcomes (*p* < 0.05), which include VAS scores, attack times, total duration, MSQ, SAS, and SDS after 4 weeks taVNS treatment (Table 1). The using dosage of the ibuprofen suspension was not normally distributed, so the Wilcoxon signed rank test was used, and the results indicated that there was no significant post- (median 0 ml, p25–p75 range 0–0 ml) and pretreatment (median 0 ml, p25–p75 range 0–10 ml) difference (*p* > 0.05) in the dosage of ibuprofen suspension for migraine patients (see Supplementary Table 1).

### Intergroup and Intragroup Comparison of Fractional Amplitude of Low Frequency Fluctuation

**FIGURE 3:**
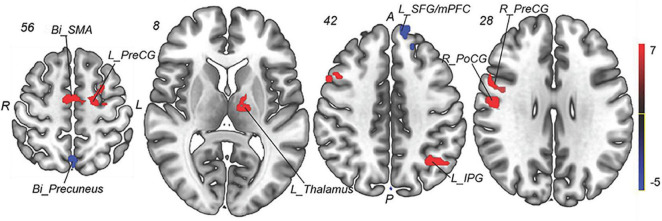
Group comparison of fALFF between the MWoA patients and HCs. Compared with HCs, pretreatment MWoA patients exhibited increased fALFF in the left thalamus, left IPG, bilateral PreCG, right PoCG, and bilateral SMA, but decreased in the bilateral precuneus and left SFG/mPFC. Red colors indicate regions with increased fALFF; blue colors indicate regions with decreased fALFF. *Bi*, bilateral; *R*, right; *L*, left; *PoCG*, postcentral gyrus; *PreCG*, precentral gyrus; *SPG*, superior parietal gyrus; *SMAs*, supplementary motor areas; *SFG*, superior frontal gyrus; *mPFC*, medial prefrontal cortex; *IPG*, inferior parietal gyrus.

**FIGURE 4:**
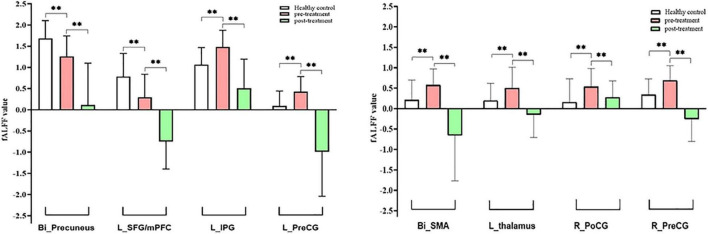
Intergroup and intragroup comparison of fALFF differences. Compared with HCs, pretreatment MWoA patients exhibited increased fALFF in the left thalamus, left IPG, bilateral PreCG, right PoCG, and bilateral SMA, but decreased in the bilateral precuneus and left SFG/mPFC; compared with pretreatment MWoA patients, migraineurs receiving 4-week taVNS treatment showed decreased fALFF in the left thalamus, bilateral PreCG, right PoCG, bilateral SMA, left IPG, bilateral precuneus, and left SFG/mPFC, but no significantly increased brain regions. White color represents healthy control; red color represents pretreatment migraine patients; green color represents posttreatment migraine patients. *Bi*, bilateral; *R*, right; *L*, left; *PoCG*, postcentral gyrus; *PreCG*, precentral gyrus; *SMA*, supplementary motor areas; *SFG*, superior frontal gyrus; *mPFC*, medial prefrontal cortex; *IPG*, inferior parietal gyrus. The ^**^represents that the difference is statistically significant.

To explore the underlying mechanism of pathophysiology of MWoA, we compared patients with MWoA to HCs using a two-sample *t*-test to detect significant abnormal regions which was used as a mask for subsequent analysis, with the age, sex, education level, and mean FD as covariates. A threshold of voxel-wise *p* < 0.005 uncorrected and a cluster-level *p* < 0.05 corrected by FDR was used for multiple comparison corrections. The results indicated that, compared with HCs, pretreatment MWoA patients exhibited increased fALFF in the left thalamus, left inferior parietal gyrus (IPG), bilateral precentral gyrus (PreCG), right postcentral gyrus (PoCG), and bilateral supplementary motor areas (SMAs), but decreased in the bilateral precuneus and left superior frontal gyrus (SFG)/medial prefrontal cortex (mPFC) (Table 2 and Figures 3, 4).

**TABLE 2:** Brain regions showing differences in fALFF for migraineurs compared to HCs.

Condition	Brain region	MNI coordinates			Peak *T-*value	Cluster
		X	Y	Z		
MWoA > HC	Left thalamus	−18	−15	12	4.25	57
	Right postcentral gyrus	45	−18	33	4.17	52
	Right precentral gyrus	54	9	30	4.42	60
	Bilateral supplementary motor areas	6	−9	57	4.62	138
	Left inferior parietal gyrus	−39	−57	39	6.71	60
	Left precentral gyrus	−29	−11	58	4.93	48
MWoA < HC	Bilateral precuneus	0	−66	54	−4.19	54
	Left superior frontal gyrus/mPFC	−12	51	42	−4.54	55

*A threshold of voxel-wise p < 0.005 uncorrected and cluster-level p < 0.05 FDR corrected was applied for second-level analyses. mPFC, medial prefrontal cortex.*

**FIGURE 5:**
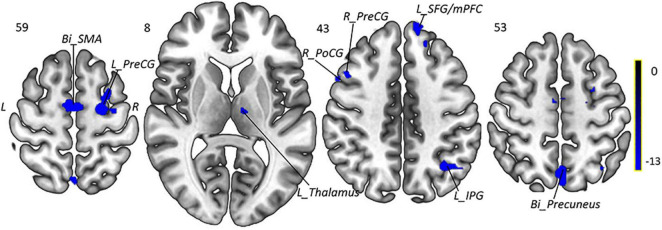
Intragroup comparison of fALFF in patients with MWoA after 4-week treatment. Compared with pretreatment MWoA patients, migraineurs receiving 4-week taVNS treatment showed decreased fALFF in the left thalamus, bilateral PreCG, right PoCG, bilateral SMA, left IPG, bilateral precuneus, and left SFG/mPFC, but no significantly increased brain regions. Blue colors indicate regions with decreased fALFF. *Bi*, bilateral; *R*, right; *L*, left; *PoCG*, postcentral gyrus; *PreCG*, precentral gyrus; *SMA*, supplementary motor areas; *SFG*, superior frontal gyrus; *mPFC*, medial prefrontal cortex; *IPG*, inferior parietal gyrus.

Paired *t*-tests were performed to compare post- and pretreatment fALFF differences for migraines with the voxel-wise *p* < 0.001 uncorrected and cluster-level *p* < 0.05 FWE corrected, and the mean FD and medication use dosage (ibuprofen suspension) were used as covariates. We observed the significant decreased fALFF in the thalamocortical (TC) circuits and default mode network after 4-week taVNS treatment. These regions were located in the left thalamus, bilateral PreCG, right PoCG, bilateral SMA, left IPG, bilateral precuneus, and left SFG/mPFC (Table 3 and Figures 4, 5).

**TABLE 3:** Brain regions showing differences in fALFF for patients between pre- and posttreatment.

Condition	Brain region	MNI coordinates			Peak *T-*value	Cluster
		X	Y	Z		
Post < Pre	Left thalamus	−9	−15	9	−8.38	12
	Right postcentral gyrus	57	0	33	−8.27	31
	Bilateral precentral gyrus	51	9	39	−12.94	41
	Bilateral supplementary motor areas	3	−9	66	−8.13	43
	Left inferior parietal gyrus	−33	−60	51	−11.31	38
	Left precentral gyrus	−24	−11	56	−11.59	62
	Bilateral precuneus	6	−60	66	−8.16	52
	Left superior frontal gyrus/mPFC	−18	36	45	−9.68	48
Post > Pre	No regions survive the threshold					

*A threshold of voxel-wise p < 0.001 uncorrected and cluster-level p < 0.05 FWE corrected was applied for second-level analyses. mPFC, medial prefrontal cortex.*

### Relationship Between Fractional Amplitude of Low Frequency Fluctuation Measures and Clinical Variables

**FIGURE 6:**
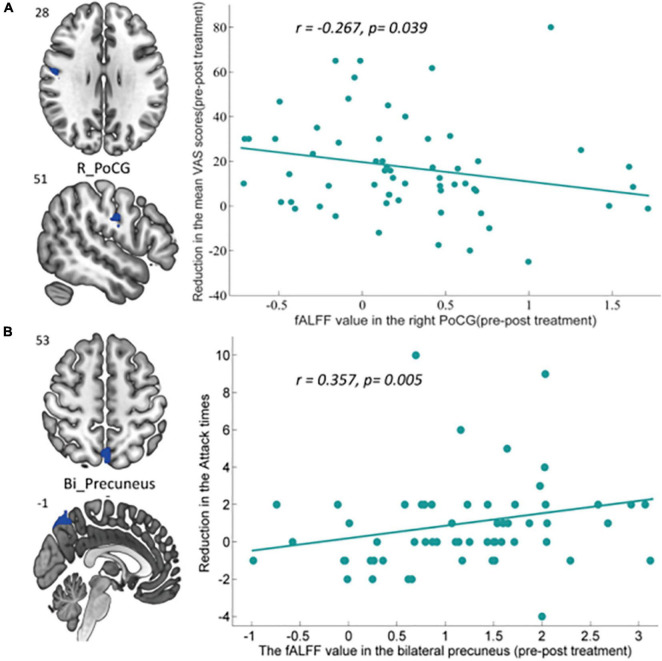
Significant correlation between the altered intragroup fALFF and clinical outcomes. **(A)** Correlation between the altered fALFF in the right PoCG (pre–posttreatment) and the reduction of VAS scores. The decreased fALFF in the right PoCG after 4-week taVNS treatment was negatively associated with corresponding reduction of VAS scores in MWoA patients (*r* = –0.267, *p* = 0.039, uncorrected). **(B)** Correlation between the altered fALFF in the bilateral precuneus (pre–posttreatment) and the reduction of migraine attack times. The decreased fALFF in bilateral precuneus after 4-week taVNS treatment was positively associated with the reduction of attack times (*r* = 0.357, *p* = 0.005, Bonferroni correction, 0.05/5). *Bi*, bilateral; *L*, left; *PoCG*, postcentral gyrus.

We performed the correlation analysis between altered regions showing significant fALFF changes in the intragroup comparison and corresponding clinical outcomes, with Bonferroni correction applied for multiple comparisons. Correlation analysis indicated that the pre- and posttreatment fALFF changes in the right PoCG after taVNS treatment were negatively associated with corresponding reduction of VAS scores in MWoA patients (*r* = −0.267, *p* = 0.039, uncorrected), and the decreased fALFF in the bilateral precuneus was positively associated with the reduction in the attack times (*r* = 0.357, *p* = 0.005) across all subjects after Bonferroni correction (0.05/5). The detailed correlation analysis results are shown in Figure 6.

### Subject-Level Prediction of Transcutaneous Auricular Vagus Nerve Stimulation Efficacy

**FIGURE 7:**
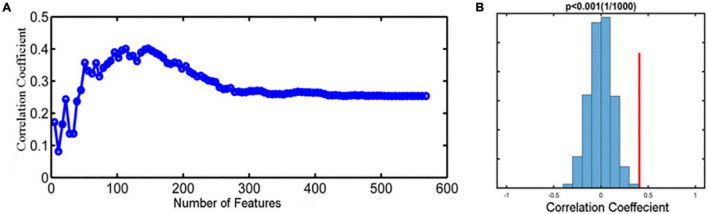
Prediction performance for taVNS treatment. **(A)** The feature selection curve reflected the changing prediction-outcome correlation following the increased number of features, and the highest correlation coefficient is 0.411. **(B)** The histogram showed the result of permutation test with highest correlation coefficient, reflecting the stability of the SVR model (*p* < 0.001).

We selected the regions indicating significant changes between pretreatment MWoA patients and HCs as our mask to predict treatment responses of taVNS (reduction of VAS scores) based on a SVR model for MWoA patients. As shown in Figure 7A, the prediction results showed that the baseline fALFF features could effectively predict the VAS score changes after 4-week taVNS treatment with a relatively high prediction-outcome correlation of 0.411. In addition, the permutation tests showed that the results could not be obtained by chance (*p* < 0.001) (Figure 7B).

**FIGURE 8:**
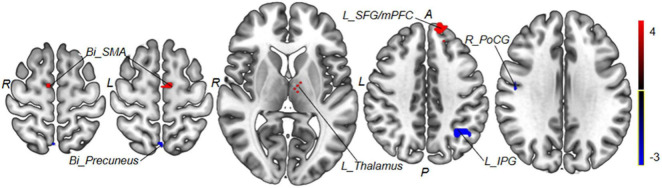
Regions with the most predictive features. Positive weight brain areas with strong predictive power are located in the left thalamus, bilateral SMA, and left SFG/mPFC; negative weight brain areas with strong predictive power are located in the right PoCG, left IPG, and bilateral precuneus. Red colors indicate regions with positive weight; blue colors indicate regions with negative weight. *Bi*, bilateral; *R*, right; *L*, left; *PoCG*, postcentral gyrus; *SMA*, supplementary motor areas; *SFG*, superior frontal gyrus; *mPFC*, medial prefrontal cortex; *IPG*, inferior parietal gyrus.

Those weight brain areas with strong predictive power were located in the left thalamus, bilateral SMA, right PoCG, left IPG, bilateral precuneus, and left SFG/mPFC (see Figure 8 and Table 4).

**TABLE 4:** The regions’ weight in predicting the treatment effect of taVNS in patients with MWoA.

Weight	Brain region	MNI coordinates			Peak *T-*value	Cluster
		X	Y	Z		
Positive weight	Left thalamus	−12	−6	6	1.71	5
	Bilateral supplementary motor areas	9	−6	63	2.14	24
	Left superior frontal gyrus/mPFC	−12	39	51	3.37	29
Negative weight	Right postcentral gyrus	48	−9	30	–2.4	15
	Left inferior parietal gyrus	−39	−36	36	–1.42	20
	Bilateral precuneus	0	−63	60	–1.47	17

*mPFC, medial prefrontal cortex.*

In addition, we predicted the treatment effect without using the difference which compared migraines with HCs as the preliminary feature selection and we also got similar results (see in Supplementary Figure 1 for details).

## Discussion

This study was an advanced exploration based on our previously published article which taVNS has been confirmed as an effective treatment for MWoA relieving acute pain (especially in reducing VAS scores) in migraine patients (Zhang et al., 2021). The current design enlarges the taVNS sample to predict the efficacy of taVNS before treatment at a subject level based on the SVR algorithm and rs-fMRI indicators.

To the best of our knowledge, this is the first study to predict the efficacy of taVNS treatment for patients with MWoA based on the mask of significant abnormal regions between the pretreatment MWoA and HCs using a SVR model combined with feature select of weight. Our results showed that the SVR model works well at predicting the treatment response of taVNS for MWoA patients at baseline (*r* = 0.411), and those weight brain areas are located in the left thalamus, bilateral SMA, right PoCG, left IPG, the bilateral precuneus, and left SFG/mPFC, mainly involved in the TC circuits, default mode network (DMN), and descending pain modulation system (DPMS). These findings suggested that the baseline fALFF values could serve as the potential biomarkers to predict taVNS treatment efficacy for MWoA.

As well known, the TC circuits have been documented involving in processing spatial and intensity aspects of noxious stimuli and also mediating the perception of pain (Magon et al., 2015; Amin et al., 2018; Martucci and Mackey, 2018; Ellingson et al., 2019). Specifically, first, as one key node of TC circuits, the thalamus holds an important position in our understanding of allodynia, central sensitization, and photophobia in migraineurs (Younis et al., 2019). Investigators have found alteration of thalamic FC in brain regions associated with pain modulating and pain encoding networks during migraine attacks (Amin et al., 2018). Second, another significant component in TC circuits, the sensorimotor network (SMN), including the PreCG, PoCG, and SMA, is thought to be activated by various sensory information from the somatosensory and motor cortices to modulate corresponding behaviors. For example, Zhang et al. (2017) have observed decreased spontaneous activity in the SMN and less FC between primary sensory areas and superior and inferior parietal lobes and also anterior cingulate cortex in MWoA patients compared with HCs. Additionally, as known, functional alterations in the TC circuits are involved in the development and maintenance of migraines (Magon et al., 2015; Amin et al., 2018; Martucci and Mackey, 2018). Increased fALFF in the TC circuit has been detected during interictal phase of migraine, and this increased fALFF in the thalamus was selectively associated with headache frequency (Hodkinson et al., 2016). In accordance with previous studies, our study showed that patients with MWoA exhibited increased fALFF in the left thalamus, bilateral PreCG, right PoCG, and bilateral SMA, compared with HCs. These findings emphasized and extended the predominant role of TC circuits in the neuropathological mechanism of MWoA.

Furthermore, this study also indicated the possibility of this circuit serving as a potential therapeutic target for patients with MWoA. In our study, we found that the aberrant fALFF in the right PoCG within TC circuits in MWoA patients was close to the normal level of HCs after 4-week treatment, and the altered fALFF in the right PoCG was negatively associated with the reduction of VAS scores (*r* = −0.267, uncorrected), which suggest that TC circuits could be identified as the useful biomarkers for taVNS response in patients with MWoA. This is in line with our previous study, in which decreased FC between the thalamic subregions and bilateral PoCG was observed with a significantly negative correlation to the reduction of the migraine days following taVNS treatment (Zhang et al., 2021).

More importantly, the predictive ability of TC circuits as candidate biomarkers for treatment response remained stable when we used a SVR model (*r* = 0.411). These findings revealed the utility of this machine learning approach for elucidating neural markers of treatment response based on TC pathways and allowed the personal differences to be rationalized. But interestingly, different from our results that the weight brain regions were mainly located in the bilateral SMA and right PoCG involving in the TC circuits, a recent study using SVR as algorithm reported that the zALFF of the bilateral middle occipital gyrus could effectively predict the relief of symptoms for migraine and hence performed a strong predictive power (Yin et al., 2020). The most possible explanation for this discrepancy might lie in the sample differences. For example, the participants in this study have relative lighter visual symptoms such as photophobia; another likely reason is due to the different intervention methods (acupuncture intervention vs. taVNS) which might produce diverse effect on brain activities. A large sample size and well-designed study should be implemented to investigate the more specific role of the TC circuits in MWoA patients after taVNS intervention.

In addition to the TC circuits, the DMN was also shown to be as the indicators of pathophysiology and treatment response for MWoA patients. Converging findings have suggested that DMN is involved in the core processes of brain function (such as internal mentation, attention, and monitoring of the surrounding environment) (Broyd et al., 2009). Disruption of the DMN has been detected in some neuropsychological disorders (Wang et al., 2013; Yin et al., 2016) and also migraine (Xue et al., 2012; Tessitore et al., 2013; Zhao et al., 2013). Consistently, in this study, we observed altered brain activities within DMN including decreased fALFF in the bilateral precuneus, left SFG/mPFC and increased fALFF in the left IPG in MWoA patients compared with HCs. What is more, as an important network closely associated with the pathophysiology of migraine, DMN is also proposed to be linked with the treatment responsiveness. As shown in our results, the increased fALFF in the left IPG in MWoA patients was normalized after effective taVNS treatment. Additionally, we found that the altered fALFF in the bilateral precuneus was positively correlated with the reduced attack times (*r* = 0.357, Bonferroni correction, 0.05/5) in MWoA patients. Coincidently, a recent acupuncture study demonstrated that the reduced FC in the left superior prefrontal cortex and precuneus in migraine patients returned to the normal level of HCs, and this alteration was associated with the alleviation of headache intensity after treatment (Zou et al., 2019). Thus, we speculated that the DMN could be used as imaging biomarkers for understanding the therapeutic mechanisms and further predicting treatment outcomes for MWoA.

Consistent with such assumption, the SVR model in this study showed that the DMN contributed much to predict the efficacy of taVNS. Interestingly, Tu et al. (2020) employed a MVPA approach together with abnormal FC within the DMN as the features to test the predictive capacity of DMN for MWoA patients, and the results demonstrated the potential role of this network for the prediction of treatment response. Similarly, another important application of machine learning showed that the FC between mPFC and subcortical regions could significantly predict therapeutic outcomes following 4-week acupuncture treatment for patients with chronic low back (Tu et al., 2019). To some extent, these findings may hint that the DMN could be used as a robust biomarker for treatment outcomes. Further investigations using different interventions are needed to validate the strong performance of DMN in patients with MWoA.

Notably, mPFC, which is not only a crucial component of DMN, but also involved in the DPMS (Ong et al., 2019), possessed high predictive weight in treatment responsiveness of taVNS in our study. As well known, the DPMS played an essential role in pain modulation and the physiopathology of chronic pain (Fields, 2004; Borsook and Burstein, 2012; Ossipov et al., 2014). Neuroimaging researches have demonstrated that chronic pain may be associated with alterations in multiple brain networks, such as the DMN, SMN, and DPMS (Kucyi and Davis, 2015, 2017). These particular networks, involved in the cognitive, sensorimotor, and affective aspects of pain, have been implicated in the core symptomatology of chronic pain and treatment response (Shi et al., 2015; Wu et al., 2016; Low et al., 2017; Lee et al., 2019; Zhang et al., 2019b). For instance, migraine patients exhibited reduced FC between the mPFC/rACC and periaqueductal gray (PAG), another key region in the DPMS compared with HCs (Li et al., 2016). More importantly, impaired brain function within the DPMS could be normalized after effective acupuncture treatment, and these changes of FCs were associated with alleviation of headache intensity. Similarly, in our study, we found decreased fALFF in the mPFC after 4-week taVNS treatment for MWoA patients, and in the following MPVA, we also revealed mPFC as an important brain region to predict the treatment responses before clinical intervention. Our findings provide potential evidence suggesting that taVNS may relieve MWoA symptoms through the mPFC-related DMN and DPMS, further which could be taken as a promising biomarker for taVNS treatment.

### Limitations

Limitations of this study should be noted. First, the sample size was relatively small. Although we did not perform the sample size estimation, we determined it according to the previously published similar article (Yin et al., 2020). Further larger scale study will be conducted to enhance the data reliability. Second, we did not take into account gender difference in our MVPA study. Considering there are less male migraines in our data, we have not further analyzed the sex difference. It is a very good idea to explore the effect of gender in migraine patients, and we will further explore it in the follow-up study. Third, patients were imaged during the interictal phase of migraine (not within 48 h before and after migraine attack or headache attack during the experiment). Larger sample size with MWoA patients at different phases such as ictal and interictal period should be designed to enhance the data integrity and reliance in the future study. Fourth, our patients were all from one single center, and the model was not externally validated. Independent and multicenter imaging datasets will be necessary to confirm our results. Finally, in the current MVPA study, only fALFF features were performed to achieve the prediction. Future studies should utilize multiple imaging modalities including structural and functional MRI analysis to validate our findings.

## Conclusion

In summary, this study preliminarily demonstrated that fALFF features at baseline have good potential for predicting individualized treatment response of taVNS for MWoA patients, and those weight brain areas are mainly involved in the TC circuits, DMN, and DPMS. This will contribute to well understanding the mechanism of taVNS in MWoA and may help to screen ideal patients who respond well to taVNS treatment.

## Data Availability Statement

The raw data supporting the conclusions of this article will be made available by the authors, without undue reservation.

## Ethics Statement

The studies involving human participants were reviewed and approved by the Ethics Committee of Guangdong Provincial Hospital of Chinese Medicine. The patients/participants provided their written informed consent to participate in this study.

## Author Contributions

BL, MF, and YZ designed the study. MF and YZ acquired the data. YZ, MF, BL, ZW, and XH performed the data analysis. MF, ZW, YZ, and YY interpreted the results. MF, BL, YZ, ZW, CF, and WL prepared the manuscript. All the authors contributed to manuscript revision and approved the submitted version.

## Conflict of Interest

The authors declare that the research was conducted in the absence of any commercial or financial relationships that could be construed as a potential conflict of interest.

## Publisher’s Note

All claims expressed in this article are solely those of the authors and do not necessarily represent those of their affiliated organizations, or those of the publisher, the editors and the reviewers. Any product that may be evaluated in this article, or claim that may be made by its manufacturer, is not guaranteed or endorsed by the publisher.

## Footnotes

1
http://www.fil.ion.ucl.ac.uk/spm

2
https://www.csie.ntu.edu.tw/c̃jlin/libsvm/index.html

## Supplementary Material

Supplementary Figure 1 Prediction of the treatment effect without using the difference which compared migraines with HCs as the preliminary feature selection (*r* = 0.392, permutation *p* = 0.0004).
