# Supplementary figures and images for: Early Fractional Amplitude of Low Frequency Fluctuation Can Predict the Efficacy of Transcutaneous Auricular Vagus Nerve Stimulation Treatment for Migraine Without Aura
Source: Front Mol Neurosci. 2022 Feb 24;15:778139. doi: 10.3389/fnmol.2022.778139 (PMC8908103; doi:10.3389/fnmol.2022.778139)

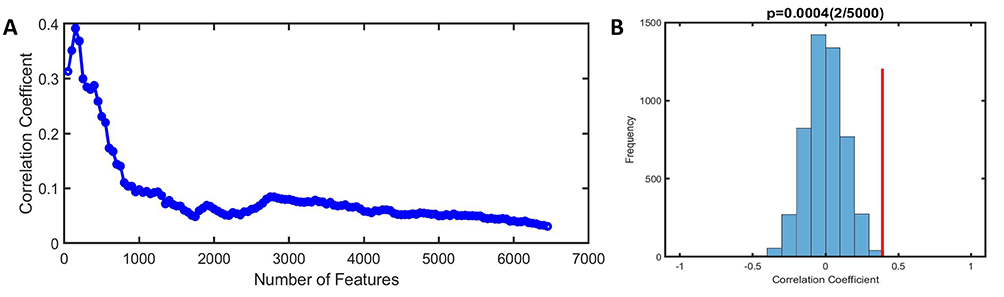

Supplement: Supplementary Figure 1 — Prediction of the treatment effect without using the difference which compared migraines with HCs as the preliminary feature selection (r = 0.392, permutation p = 0.0004). [file Image_1.tif]
