# Supplementary material for: Early Fractional Amplitude of Low Frequency Fluctuation Can Predict the Efficacy of Transcutaneous Auricular Vagus Nerve Stimulation Treatment for Migraine Without Aura
Source: Front Mol Neurosci. 2022 Feb 24;15:778139. doi: 10.3389/fnmol.2022.778139 (PMC8908103; doi:10.3389/fnmol.2022.778139)
Supplement: Supplementary file 2 [file Table_1.docx]

| **TABLE 1\| Using dosage of ibuprofen suspension before treatment and after treatment.** | | | |
| --- | --- | --- | --- |
|  | Pre-treatment | Post-treatment | *P* |
| Using dosage of the ibuprofen suspension(ml) | 0(0,10) | 0(0,0) | 0.401 |

The using dosage of ibuprofen suspension before treatment and after treatment were not normally distributed. Wilcoxon signed ranks test results indicated that there was no significant post- treatment(median 0ml, p25-p75 range 0-0ml) and pre- treatment(median 0ml, p25-p75 range 0-10ml) difference(*p*>0.05) in the dosage of ibuprofen suspension for patients.
